# Lung Carcinoids—Time to Change Practices

**DOI:** 10.3390/curroncol33010050

**Published:** 2026-01-15

**Authors:** Ana Rodrigues, Nuno Coimbra, Inês Lucena Sampaio, Isabel Azevedo, Marta Soares, Carmen Jerónimo, Rui Henrique

**Affiliations:** 1 Department of Medical Oncology, Portuguese Oncology Institute of Porto, 4200-072 Porto, Portugal; isabel-azevedo@ipoporto.min-saude.pt (I.A.); marta.soares@ipoporto.min-saude.pt (M.S.); 2 Thoracic and Mediastinal Tumors Clinic, Portuguese Oncology Institute of Porto (IPO Porto)/Porto Comprehensive Cancer Center Raquel Seruca (Porto.CCC), 4200-162 Porto, Portugal; nuno.coimbra@ipoporto.min-saude.pt; 3 Department of Pathology, Portuguese Oncology Institute of Porto (IPO Porto)/Porto Comprehensive Cancer Center Raquel Seruca (Porto.CCC), 4200-162 Porto, Portugal; henrique@ipoporto.min-saude.pt; 4 Cancer Biology and Epigenetics Group, Research Center of IPO Porto (CI-IPOP) IPOP/CI-IPOP@RISE (Health Research Network), Portuguese Oncology Institute of Porto (IPO Porto)/Porto Comprehensive Cancer Center Raquel Seruca (Porto.CCC Raquel Seruca), 4200-162 Porto, Portugal; carmenjeronimo@ipoporto.min-saude.pt; 5 Department of Nuclear Medicine, Portuguese Oncology Institute of Porto (IPO Porto)/Porto Comprehensive Cancer Center Raquel Seruca (Porto.CCC), 4200-162 Porto, Portugal; ines.lucena@ipoporto.min-saude.pt; 6 Department of Pathology and Molecular Immunology, ICBAS-School of Medicine & Biomedical Sciences, University of Porto, 4050-313 Porto, Portugal

**Keywords:** real-world data, retrospective study, lung carcinoids, neuroendocrine tumors

## Abstract

**Background**: Lung carcinoids—typical and atypical—are rare neuroendocrine tumors (NETs) representing 1–2% of lung cancers. Despite clinicopathological differences, their clinical management often mirrors lung cancer protocols rather than NET-specific recommendations. **Objectives**: Portray a 12-year real-world experience with lung carcinoids at a Comprehensive Cancer Center, identifying gaps in diagnostic work-up, treatment decision-making, and follow-up. **Methods**: Retrospective observational cohort study of adult patients with histologically confirmed lung carcinoids diagnosed at IPO Porto between January 2013 and December 2024. Demographic, clinical, imaging, and treatment data were collected from electronic patient records. Analyses were descriptive. **Results**: Among 179 identified cases, 129 met eligibility criteria. Median age was 62 years (range 18–84); 53.6% were women and 53.5% were non-smokers; 84.5% had ECOG-PS 0–1. The most frequent presentation was respiratory symptoms (34.1%), followed by incidental findings (43.4%, of which ~20% were during staging or surveillance of other cancers). Typical carcinoids accounted for 49.6% and atypical for 43.4%. FDG-PET/CT was requested in 70.9% of cases, including many with typical carcinoid, and SSTR-PET/CT in 64.6% (dual PET in 38.8%). Most patients (65.1%) presented with stage I disease; 17.1% were stage IV. Mean time-to-first treatment was 83 days (range 1–259). Surgery was the first treatment option for 78.3% of patients. **Conclusions**: This real-world series highlights heterogeneity in diagnostic pathways, excessive FDG-PET use in typical carcinoids, and non-standardized follow-up. Dedicated multidisciplinary lung-NET boards and national reference centers are needed to homogenize and streamline patient management.

## Simple Summary

Lung carcinoids—typical and atypical—are rare neuroendocrine tumors, whose management often mirrors lung cancer protocols rather than NET-specific recommendations. Our retrospective evaluation of 12-year real-world experience with lung carcinoids at a Comprehensive Cancer Center identifies gaps in diagnostic work-up, treatment decision-making, and follow-up, such as excessive FDG-PET use in typical carcinoids. This finding reinforces the need for dedicated multidisciplinary lung-NET boards and national reference centers to homogenize and streamline patient management.

## 1. Introduction

Lung carcinoid tumors—typical or atypical—are neuroendocrine tumors (NETs) arising from bronchopulmonary neuroendocrine cells. They present an incidence of 0.2 to 2 cases per 100,000 inhabitants worldwide, representing 1–3% of lung cancers and 30% of all NETs [1,2].

Their incidence is rising, likely due to improved diagnostics and awareness among clinicians and pathologists, although environmental and/or genetic factors may also contribute [1,3,4,5]. These tumors are generally slow-growing, with 80–95% presenting localized disease. Nevertheless, even after surgery, the 5-year survival rate for atypical carcinoids rarely exceeds 50–70% [6]. Patients are often younger than those with lung cancer and usually present with respiratory symptoms; some, however, are found incidentally through imaging [7].

Due to their low incidence, there are several gray areas in the management of lung carcinoids. For instance, there is a dichotomous histological classification into typical and atypical carcinoids, relying solely on necrosis and mitotic count, whereas in gastroenteropancreatic (GEP) NETs, there is a three-tiered system that incorporates proliferation index (through Ki67 immunostaining), better reflecting tumor biology [8]. Another aspect concerns the lack of clinical trials dedicated to this pathology, with existing evidence originating from retrospective case series and subgroup analyses of patients with lung NETs included in prospective trials for NETs. This limits basic, translational, and clinical research in this context and negatively affects the management of these patients. Indeed, patients are often managed heterogeneously, frequently making use of the principles of lung cancer care [9].

Historically, decisions and treatment were managed by thoracic surgeons or, in general, thoracic tumor boards, without NET experts’ input, but there is an increasing trend toward Multidisciplinary Tumor Boards (MDTBs) dedicated to NET [10]. In-depth analysis of current clinical practices is key to identifying gaps and unmet needs that may inform the development and implementation of lung NET-specific clinical pathways. Thus, we sought to review the practice and experience in lung NET patient management at a tertiary, cancer-dedicated center—Portuguese Oncology Institute of Porto/Porto Comprehensive Cancer Center (IPO Porto/Porto.CCC)—between January 2013 and December 2024, providing a critical analysis of the clinical approach and management of these patients.

## 2. Materials and Methods

### 2.1. Study Design

Observational, retrospective, cohort, single-center, secondary-use data study including patients with lung carcinoids diagnosed between 1 January 2013 and 31 December 2024, at IPO Porto/Porto.CCC. For each patient, data were retrospectively retrieved from the date of admission until the patient’s last contact, death, or data cut-off. The study database was locked in August 2025. This study was approved by the Ethics Committee of IPO Porto/Porto.CCC (CES.060_25_A_016_25). Patients’ informed consent was waived due to the retrospective observational nature of this study and data anonymization.

### 2.2. Selection of Patients

Eligible patients were male or female, aged 18 years or older, with histologically or cytologically confirmed lung carcinoid (typical, atypical, or carcinoid NOS), diagnosed between January 2013 and December 2024. Patients were excluded if: they had received treatments outside IPO Porto at any time; early loss to follow-up (<6 months) after the first therapeutic approach (for example, cases of inter-hospital collaboration protocols and discharge from the institution after surgery); loss of follow-up due to patient’s convenience in changing hospitals; history of synchronous or metachronous cancers (<5 years) that were metastatic at diagnosis or presented disease progression during follow-up, directly compromising lung NET treatment and patient survival; NET in the context of diffuse idiopathic pulmonary neuroendocrine cell hyperplasia (DIPNECH), suspected radiologically or confirmed histologically in the surgical specimen. Patients with diffuse idiopathic pulmonary neuroendocrine cell hyperplasia (DIPNECH) and those with advanced synchronous malignancies were excluded in order to minimize confounding factors affecting diagnostic pathways, multidisciplinary decision-making, and follow-up strategies. As the primary aim of this study was to characterize real-world clinical pathways rather than treatment outcomes, inclusion was restricted to cases in which lung carcinoid represented the main driver of clinical management.

### 2.3. Data Collection

Demographic, clinical, and treatment data were extracted from IPO Porto’s electronic medical and administrative records. Staging was re-coded using the 9th Edition of the TNM Classification for Lung Cancer [11].

Confidentiality and anonymization of the data for analysis were strictly maintained. Data linkage between sources was accomplished using the unique internal identifier assigned by IPO Porto upon admission.

### 2.4. Outcomes

Follow-up time was defined as the time from diagnosis to last observation or death. Time to first treatment was defined as the time from the diagnosis to the first treatment performed, whatever its nature (surgery, radiotherapy, or systemic treatment). Main descriptive outcomes included distribution by stage/histology, initial MDTB pathway, imaging modality, time-to-first treatment (TFT), and type of first treatment.

Real-world outcomes, such as progression-free survival, overall survival, time on treatment, or time to next treatment, were not evaluated due to the small number of events (progression or death).

### 2.5. Statistical Analysis

All patients at IPO Porto fulfilling the eligibility criteria were included in the statistical analysis (no formal sample size calculation was performed). Statistical analysis was conducted for the overall sample. Demographic, clinical, and treatment characteristics were summarized using descriptive statistics.

The statistical analysis was performed using Microsoft Office Excel 2019 version 1808 and R software version 4.0.5.

## 3. Results

Of 179 patients diagnosed with lung carcinoid, aged 18 or older, between 2013 and 2024, 129 were included in this analysis, and 50 were excluded. Exclusion reasons were: 12 received treatment outside the institution; 13 were lost to follow-up due to transfer, discharge post-surgery, or transfer to another institution; 16 had metastatic cancer affecting lung NET management and survival; and 9 had lung carcinoid associated with DIPNECH, identified radiologically or histologically.

**Figure 1:**
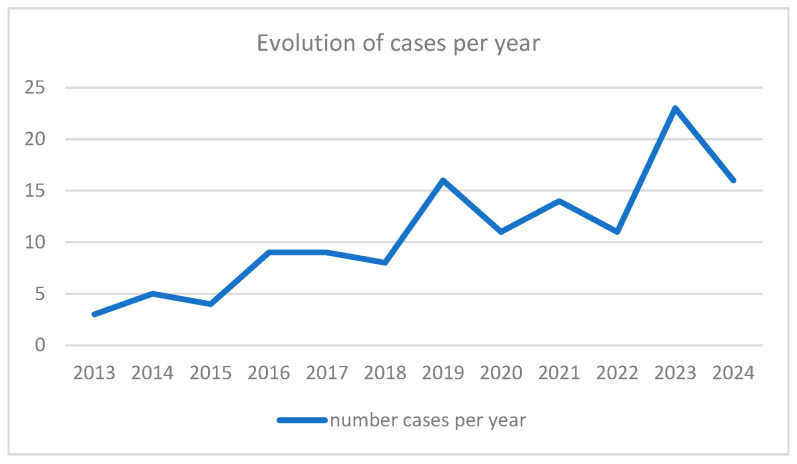
Evolution of the number of cases per year.

Since 2019, there has been a considerable increase in the number of cases per year (Figure 1).

Median age at diagnosis was 62 years (minimum 18 and maximum 84 years), 53.59% were female, 53.49% were non-smokers, and most of the patients presented ECOG PS (Eastern Cooperative Oncology Group Performance Scale) 0–1. The most frequent presentation was respiratory symptoms, including recurrent infections (34.11%), followed by pain (4.65%) and constitutional symptoms (asthenia, anorexia, and weight loss) (8.53%, overall). Incidental radiological findings represented a significant proportion of the cases (43.41%), with almost half of them (20.15%) during staging or surveillance of other cancers. Typical carcinoids and atypical carcinoids represented 49.61% and 43.41% of the population, respectively, and only in 5.53% of cases was subtype discrimination not possible (Table 1).

A significant percentage of patients (50 (34.88%)) had a history of another malignancy, and among these, 62.22% were diagnosed with a synchronous neoplasm or within less than 5 years. The most frequent malignancies were from breast (13 patients), prostate (6), lung (4), endometrium (3), and GEP-NET (3), among others.

At IPO Porto, the initial appointment was in the Thoracic and Mediastinal Tumors Clinic for all patients, including those already diagnosed with lung carcinoid (57 pts (44.18%)). All first appointments were made by clinicians dedicated to thoracic oncology, such as medical oncologists (67 (51.94%)), thoracic surgeons (40 (31.01%)), or pneumologists (22 (17.05%)).

The staging exams requested were FDG-PET/CT in 70.87% of cases and SSTR-PET/CT in 64.57%, with 38.76% undergoing dual evaluation by PET. Of the 57 patients admitted with a biopsy diagnosis of lung carcinoid, 50 were assessed by dual PET. Among these, 23 had a preoperative biopsy diagnosis of atypical carcinoid and 24 of typical carcinoid. The remaining 3 patients assessed by dual PET correspond to those with pulmonary lesions under investigation without biopsy results.

The number of patients who underwent brain assessment by Computerized Tomography (CT) or Magnetic Resonance Imaging (MRI) was residual, and the evaluation of chromogranin A and 5-HIAA was performed in 22.48% and 20.93% of cases, respectively.

Most patients (65.12%) had stage I disease, 13.95% had stage II, 3.88% had stage III, and 17.05% had stage IV disease. The mean time of follow-up was 3.64 years, and the median was 2.88 years. The mean time for the first treatment was 83 days (range 1 to 259 days). Four patients did not receive any treatment and were assigned to either surveillance or best supportive care.

Most patients were discussed in MDTBs before any treatment, mainly the Thoracic MDTB (86 patients), followed by the Endocrine MDTB (29 patients). Patients with stage IV disease were preferably discussed in the Endocrine MDTB. The therapeutic decisions included surgery of the primary tumor, systemic treatment (Chemotherapy and/or Somatostatin Analogs (SSAs)), best supportive care, clinical vigilance, concomitant chemoradiotherapy, and stereotactic radiotherapy (Table 2). In 12 cases, patients were submitted to surgery without a previous decision in an MDTB.

Out of the 129 patients, 103 underwent surgery for the primary tumor. Of these, 62.14% had a lobectomy with lymph node dissection, 17.48% underwent wedge resection with lymph node dissection, 14.56% had either wedge resection or lobectomy without lymph node assessment, 3.88% underwent pneumonectomy, and 1.94% had other procedures.

At the time of the cut-off of the dataset, only 24 events (deaths) had occurred, 13 of them in patients with stage IV disease at diagnosis. Among patients treated with curative-intent surgery, only 6 disclosed disease progression, mainly locoregional, bone, and/or hepatic.

Follow-up of patients submitted to surgery was mainly accomplished by medical oncology (59%), followed by thoracic surgery. All included history and physical examination, but the selected imaging method varied considerably. Medical oncology used thoracic CT, while thoracic surgery alternated between CT and SSTR-PET/CT. Both, however, followed patients with the same periodicity as lung cancer patients, and frequently, patients were discharged after 5 years of uneventful follow-up.

## 4. Discussion

In this study, we analyzed the clinical pathway of patients diagnosed with lung NETs at IPO Porto/Porto.CCC between 2013 and 2024. Our main goal was to portray the current clinical practice, identifying gaps and unmet needs, enabling the implementation of an improvement plan that may increase effectiveness and sustainability in the provision of care for those patients.

Following the same trend observed worldwide, we observed a considerable increase in the number of diagnosed cases of lung NET since 2019. Multiple factors may have contributed to this, including a progressive increase in imaging exams performed in the context of benign pathology, the COVID-19 pandemic, and growing awareness regarding lung cancer screening.

Because this analysis focused on organizational pathways rather than biological heterogeneity or treatment efficacy, a degree of cohort refinement was necessary. In this context, the exclusion of patients with diffuse idiopathic pulmonary neuroendocrine cell hyperplasia (DIPNECH) and those with advanced synchronous malignancies aimed to minimize background noise and ensure interpretability of diagnostic work-up, multidisciplinary decision-making, and follow-up strategies specifically related to lung carcinoids as the primary disease.

As the institution is organized according to pathology units and does not have a unit dedicated solely to NET, almost all patients, including those already diagnosed with lung carcinoid (44.18%), were admitted through the Thoracic and Mediastinal Tumors Clinic, which has important implications. One of these is the staging assessments requested, since patients follow the pathway defined for common lung cancers. Hence, many patients underwent FDG-PET/CT evaluation, including those with previously identified typical carcinoids. Current guidelines recommend staging with chest and abdominal CT and suggest SSTR-PET/CT and brain MRI only if clinically indicated. While brain metastases are rare in typical carcinoids, their incidence increases in atypical carcinoids, particularly in tumors with higher proliferative indices, supporting a more selective but proactive approach to central nervous system imaging in this subgroup. Regarding FDG-PET/CT, it is not recommended for routine use. Combined assessment using FDG-PET/CT and SSTR-PET/CT may be appropriate for atypical carcinoid cases, high-grade histopathology, or negative SRI, to better assess the biological behavior [9,10,12,13]. Thus, our study clearly identifies an overuse of PET in an era in which diagnostic testing should be managed carefully due to increasing demands and limited resources.

Another implication is the MDTB, in which lung NETs are discussed, and the composition of each MDTB. The Thoracic MDTB includes a dedicated thoracic radiotherapist and medical oncologist, pneumologist, thoracic surgeon, and radiologist, and the main pathology discussed is lung cancer. The endocrine MDTB includes a medical oncologist, endocrinologist, nuclear medicine specialist, and general surgeons, and the main pathologies discussed are thyroid cancers and GEP-NET. Lung NETs are discussed in both MDTBs, although mostly in the thoracic MDTB, a reality that is transversal to many institutions worldwide. Nonetheless, it is widely acknowledged that adequate and homogenous management of these patients requires that decisions be taken at the same MDTB. Multidisciplinary teams specifically dedicated to NETs constitute, indeed, an unmet need. While clinical practice guidelines have their merits, centralization of management, particularly for complex cases involving metastatic disease, G2 tumors, or carcinoid syndromes, must be considered [9].

Finally, the organization of the institutions has an impact on the follow-up of these patients. The optimal follow-up protocol is not well established due to limited evidence and the generally indolent nature of these tumors. The frequency and duration of follow-up visits may vary based on individual patient characteristics and the specific features of the tumor. According to the currently available ESMO guidelines, the duration of follow-up for lung carcinoids should be lifelong, as over time, recurrences may occur. Nonetheless, this is not evidence-based and has not been shown to be associated with a survival benefit [12]. The NCCN recommends follow-up assessments at 12 weeks and 12 months following surgical intervention. These evaluations should include a comprehensive history and physical examination, chest and abdominal CT scans with contrast, and biochemical markers for functional tumors, as clinically appropriate. Further assessments are advised every 12 to 24 months until the tenth year post-surgery, with continued surveillance thereafter as indicated. Guideline recommendations for SSTR-PET/CT surveillance in resected pulmonary carcinoids are highly heterogeneous: the European Neuroendocrine Tumors Society (ENETS) suggests imaging every 12–24 months, ESMO recommends follow-up only in cases of doubtful or abnormal imaging, whereas the Commonwealth Neuroendocrine Tumour Research Collaboration (CommNETS), paired with the North American Neuroendocrine Tumor Society (NANETS) and NCCN, does not routinely endorse it [12,13,14].

The follow-up at most institutions, including IPO Porto, differs significantly from the recommendations above. For instance, by following the same pathway as lung cancer, the number of appointments and imaging evaluations increases, and the natural history of the disease is not considered, resulting in frequent discharge of the patient after a follow-up of 5 years. However, the main criticism of the follow-up procedure is the inappropriate use of SSTR-PET/CT scans alternating with CT in the monitoring of these patients. This could be solved by centralizing follow-up in one or two dedicated experts, as well as by establishing follow-up guidelines and conducting outcome audits.

Retrospective studies are generally of limited value but remain important for rare diseases due to scarce evidence, and this is the key strength of this report. This study was not designed to evaluate the efficacy of systemic therapies or long-term oncological outcomes, given the small number of events and its descriptive focus on clinical pathways. These aspects warrant dedicated future analyses in larger, preferably multicenter cohorts. Recognizing their significance, ENETS launched in July 2021 the Lung NET Task Force, aiming to collect larger patient series and generate stronger evidence [15]. The task force joins international experts from various disciplines to collaborate on retrospective and translational lung NET projects. Another important point is the awareness that practice changes, orientations, and harmonization of the management of these patients are an unmet clinical need.

Currently, Portugal has no ENETS-certified centers, but we must work towards the necessary restructuring and training of health professionals, so that national oncology centers may become candidates for that certification.

## 5. Conclusions

This real-world series provides an in-depth overview of the management of lung carcinoid tumors over a 12-year period in a Comprehensive Cancer Center. Despite the overall favorable prognosis of these rare tumors, our findings highlight a substantial heterogeneity in diagnostic and follow-up strategies, reflecting the lack of consensus and dedicated clinical pathways. The frequent use of FDG-PET/CT even in typical carcinoids, and the absence of standardized follow-up schedules tailored to tumor biology, underscore the need for evidence-based, harmonized management protocols.

The standardization of criteria for the approach and follow-up of pulmonary carcinoid tumors, as exemplified by the work of the ENETS lung NET task force, is essential to ensure that diagnostic and therapeutic decisions are aligned with the unique biological behavior of lung neuroendocrine tumors. The establishment of national reference centers and dedicated MDTBs for lung NETs would promote uniform care, facilitate research collaboration, and enable audit of outcomes at a population level.

From a healthcare resources and policy perspective, our study underscores the importance of rational resource allocation in the era of escalating imaging costs and constrained nuclear medicine capacity. The widespread and often unnecessary use of dual PET imaging in typical carcinoids exemplifies how unstandardized practices may negatively impact and strain healthcare systems without clear benefit. National policies should therefore promote cost-effective diagnostic algorithms and centralized case discussions to optimize resource utilization, particularly in countries with limited access to advanced imaging modalities.

In summary, integrating multidisciplinary expertise, aligning practices with international recommendations, and prioritizing cost-effective strategies will be crucial to improve the quality, consistency, and sustainability of care for patients with lung carcinoid tumors.

## Figures and Tables

**Table 1:** Demographic and clinical characteristics at diagnosis.

Variable	Number (%)
**Sex**	
Male	60 (46.51)
Female	69 (53.59)
**Smoking habits**	
Non-smoker	69 (53.49)
Ex-smoker	25 (19.38)
Smoker	14 (10.85)
Unknown	21 (16.28)
**ECOG PS**	
0–1	109 (84.50)
2–4	10 (7.75)
Unknown	10 (7.75)
**Clinical presentation**	
Respiratory symptoms	44 (34.11)
Incidental radiological finding	30 (23.26)
Surveillance of other cancers	20 (15.50)
Asthenia, anorexia, and weight loss	11(8.53)
Staging of other cancers	6 (4.65)
Pain	6 (4.65)
Flushing and diarrhea	3 (2.33)
Surgical finding	3 (2.33)
Cushing syndrome	1 (0.78)
Others or unknown	5 (6.19)
**Location**	
Right	66(51.97)
Left	57 (44.88)
Bilateral	4 (3.15)
**Histology**	
Typical carcinoid	64 (49.61)
Atypical carcinoid	56 (43.41)
Carcinoid NOS	7 (5.43)
Typical and atypical	2 (1.55)

**Table 2:** Treatment decision.

1st Treatment Decision	Number (%)
Surgery	101 (78.29)
SSA	7 (5.43)
Chemotherapy	6 (4.65)
Chemotherapy + SSA	4 (3.10)
Best supportive care	3 (2.33)
Surgery + SSA *	2 (1.55)
Vigilance	2 (1.55)
Chemoradiotherapy	2 (1.55)
Radiotherapy	2 (1.55)
Total	129 (100)

* Stage IV disease, proposed for surgery of the primary tumor and SSA.

## Data Availability

The study dataset is not publicly available due to privacy or ethical restrictions, but access may be requested from IPO Porto if reasonable and scientifically relevant.

## Abbreviations

The following abbreviations are used in this manuscript:

NETs	Neuroendocrine tumors
GEP NETs	Gastroenteropancreatic Neuroendocrine Tumors
MDTDs	Multidisciplinary Tumor Boards
CCC	Comprehensive Cancer Center
DIPNECH	Diffuse idiopathic pulmonary neuroendocrine cell hyperplasia
ECOG PS	Eastern Cooperative Oncology Group Performance Scale
SSTR-PET/CT	Somatostatin receptor PET/CT
FDG-PET/CT	18F-fluorodeoxyglucose PET/CT
CT	Computerized Tomography
MRI	Magnetic Resonance Imaging
